# Primary Hydatid Cyst in the Axillary Region: A Case Report

**Published:** 2018

**Authors:** Erdem KARADENİZ, Mesut YUR, Müfide Nuran AKÇAY, Sabri Selçuk ATAMANALP

**Affiliations:** Dept. of General Surgery, Faculty of Medicine, Ataturk University, Erzurum, Turkey

**Keywords:** Hydatid cyst, Axillary, Primary, Turkey

## Abstract

Axillary region is one of the areas where primary hydatid cyst is rare. In this study, we present a case of isolated axillary hydatid cyst in a 40 year-old female patient having applied to our polyclinic with swelling and pain on right axillary. During the breast examination of the patient on right axillary, well-circumscribed semi-mobile mass lesion was detected. On mammary ultrasonography, both breasts were natural. There was necrotic lymphadenopathy on right axillary that was roughly 10×10 cm sized, and locally included cystic patency. Axillary LAP excision was planned for histopathologic diagnosis. Patient was taken to the operation. After it was understood that cyst was hydatic, it was excised with germanium membrane by encircling it with savlon compresses. In order to differentiate primary secondary on post-operative patient, the patient was taken to thoracic and abdomen tomography. No cystic lesions were detected on tomography. Having no problems on follow-ups, the patient was discharged on 3 post-op days with recommendations, and with starting albendazole 10 mg/kg.

## Introduction

Hydatid cyst disease is one of the zoonoses caused by Echinococcus granulosus showing the wide geographic spread in the world (1). In humans, it is transmitted by animals located in the life cycle. It is common in the regions such as Mediterranean, Eastern Europe, Africa, South America, Middle East, Australia, New Zealand and China where livestock farming, particularly sheep breeding is widespread (2,3). Although hydatid cyst disease that is endemic in our country is most often seen in the liver, it may also be observed in the lungs, muscle tissue, soft tissue, kidney, spleen, bone and less frequently in brain, breast tissue, heart, paranasal sinuses (1–3).

## Case presentation

**Fig. 1:**
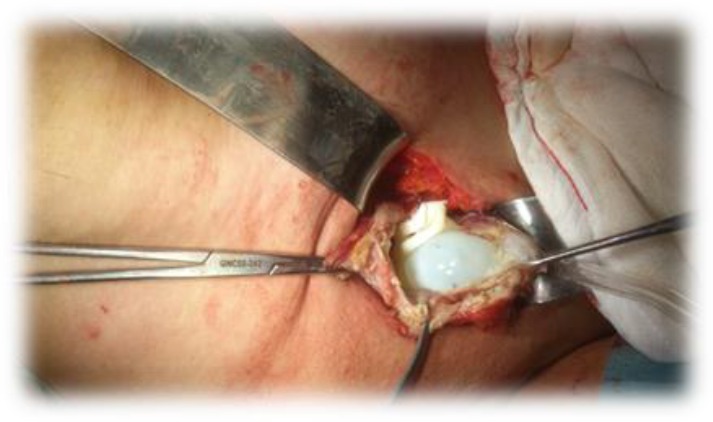
Operation of cystic mass lesion at axillary area

A 40 year-old female patient applied to our polyclinic with swelling and pain on right axillary which had been continuing for about 2 months. During the breast examination of the patient who had no breast cancer cases in her family history, no features were detected on both breasts and left axillary. On right axillary, well-circumscribed semi-mobile mass lesion was detected. No features were found on biochemical investigations. On mammary ultrasonography (USG), it was reported that both breasts were natural, and there was necrotic lymphadenopathy (LAP) on right axillary that was roughly 10×10 cm sized, and locally included cystic patency. Axillary LAP excision was planned for histopathologic diagnosis. The patient was taken to the operation. By right axillary incision, skin and subcutan were passed. Cystic mass lesion was at axillary area. While trying to take of the lesion, capsule was perforated. Rock water and female vesicles were drained out (Fig. 1).

**Fig. 2:**
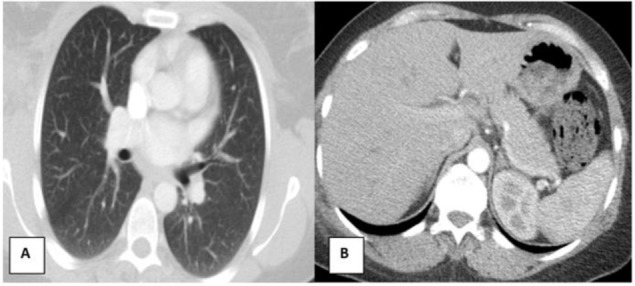
No cystic lesions were found on tomographies after operation

After it was found out that cyst was hydatic, it was excised with germanium membrane by encircling it with savlon compresses. In order to differentiate primary secondary on postoperative period, the patient was taken to thoracic and abdomen tomography. No cystic lesions were found on tomographies (Fig 2a, 2b). Having not any problems on follow-ups, the patient was discharged with recommendations, and with starting albendazol 10 mg/kg on 3 post-op days.

## Discussion

Hydatid cyst disease is still an important health issue in agricultural communities, and Turkey is one of these countries (4).

In humans, the disease is usually localized to the liver and lungs; and 13.9% of the cases have a rare localization of the hydatid cyst (5). Among these rare localizations, spleen, soft tissue, abdominal cavity, kidneys, brain, bone, pancreas, breast tissue, pelvis, joints, bladder, heart, ovaries, thyroid gland, retroperitoneum, incision scar and common bile duct can be mentioned in order of frequency(5). Primary axillary hydatid cyst is very rare even in the endemic areas (6–10).

Patients with axillary hydatid cyst are usually at the age range between 25 and 45, and they are often presented with pain and swelling at the axillary area. Our case was a 40 year-old woman. Her physical examination revealed that she had a semi-mobile palpable lump in her right axilla. Imaging methods were very helpful in the diagnosis of the disease, often revealing some specific signs of the disease. Ultrasonography, computed tomography and magnetic resonance imaging can be used to visualize the cyst’s relation to the surrounding tissues, internal luminal wall of the cyst, and intraluminal daughter cysts (11).

In our case, considering the possibility of breast cancer metastasis as the cause of the lesion, the patient was examined with bilateral breast and axillary ultrasonography. Breast and axillary ultrasonography was reported as bilaterally normal breast tissue, and necrotic lymphadenopathy at the right axillary area which included few cystic cavities. Over time, *Echinococcus granulosus* infection can lead to structural deformities in the tissue, and particularly the unilocular hydatid cysts with moderate or hyper densities can be confused with other pathologies like sarcoma or conglomerated lymphadenopathies (11–13). The PET CT examination performed to the patient in an external center did not show any features. Therefore, instead of further imaging studies, excisional biopsy was planned to diagnose the axillary mass lesion.

Serological tests may aid in the diagnosis, but serological tests have high false positivity rates (6, 9). Since we did not initially consider primary axillary hydatid cyst disease clinically or radiologically in our patient, we did not perform serological test for hydatid disease. Diagnostic aspiration cytology can also be used in the diagnosis but it introduces the risk of anaphylaxis and insemination of the organism along the aspiration line (14).

Although our region is an endemic region for hydatid cysts and frequent occurrence of hydatid cysts, it has been ruled out that the lesion might be hydatid cyst at ultrasonografi due to the rare localization of the cystic site. After the ultrasonography examination, it could be more appropriate in our case to confirm the diagnosis with an advanced imaging modality such as magnetic resonance imaging or computed tomography, and to conduct proper preoperative medical preparations.

The disease was treated with total excision of the cystic lesion following prophylaxis with albendazole. Besides, other commonly affected sites should also be investigated because of the fact that primary axillary hydatid cyst disease is extremely rare. Seeing that our case was diagnosed intraoperatively, no preoperative prophylaxis was administered. The lesion was excised totally. The patient was scanned with thoracic and abdominal tomography at the post-operative period, and her treatment was completed because any other pathology was not detected.

## Conclusion

Although primary axillary hydatid cyst is an extremely rare disease, it should be considered among the differential diagnosis for patients presented with axillary swelling symptoms in the endemic areas.
